# Disease pathogenicity in Hutchinson–Gilford progeria syndrome mice: insights from lung-associated alterations

**DOI:** 10.1186/s10020-025-01165-x

**Published:** 2025-03-24

**Authors:** Jingjing Wang, Yuelin Guan, Yue Wang, Junyi Tan, Zhongkai Cao, Yuhan Ding, Langping Gao, Haidong Fu, Xiangjun Chen, Jianyu Lin, Ning Shen, Xudong Fu, Fangqin Wang, Jianhua Mao, Lidan Hu

**Affiliations:** 1 Department of Nephrology, The Children’s Hospital, Zhejiang University School of Medicine, National Clinical Research Center for Child Health, Hangzhou, 310020 Zhejiang Province China; 2 Hubei Normal University, Huangshi, 435002 China; 3 Institute of Translational Medicine, Zhejiang University School of Medicine, 268 Kaixuan Road, Hangzhou, 310020 China; 4 Liangzhu Laboratory of Zhejiang University, Hangzhou, 310020 Zhejiang China

**Keywords:** Aging, Hutchinson–Gilford progeria syndrome, Lungs, Progerin, Rare diseases

## Abstract

**Background:**

Hutchinson–Gilford progeria syndrome (HGPS) is a rare genetic disorder characterized by accelerated aging, impaired growth, disrupted lipid metabolism, and reduced lifespan.

**Methods:**

Prior research has primarily focused on cardiovascular manifestations, our research sheds light on multiple organs that underwent significant age-related changes validated by tissue cross-sections H&E, Masson's trichrome, and β-galactosidase staining.

**Results:**

Among these pathologies tissues, the lung was severely affected and substantiated by clinical data of pulmonary anomalies from our HGPS patients. Biochemical and histological analyses of lung tissue from the HGPS mouse model revealed elevated Progerin expression, abnormal NAD metabolism, cellular senescence markers (higher level of p16 and p27, lower level of ki67), and various age-related morphology changes, including fibrosis, inflammation, and thickening of alveolar walls. Transcriptomic analyses of lung tissue indicated that down-regulated genes (*Thy1, Tnc, Cspg4, Ccr1*) were associated with extracellular space, immune response, calcium signaling pathway, osteoclast differentiation, and lipid binding pathway.

**Conclusions:**

This study unveiled the previously overlooked organs involved in HGPS pathogenesis and suggested a specific emphasis on the lung. Our findings suggest that pulmonary abnormalities may contribute to disease progression, warranting further investigation into their role in HGPS monitoring and management.

**Graphical abstract:**

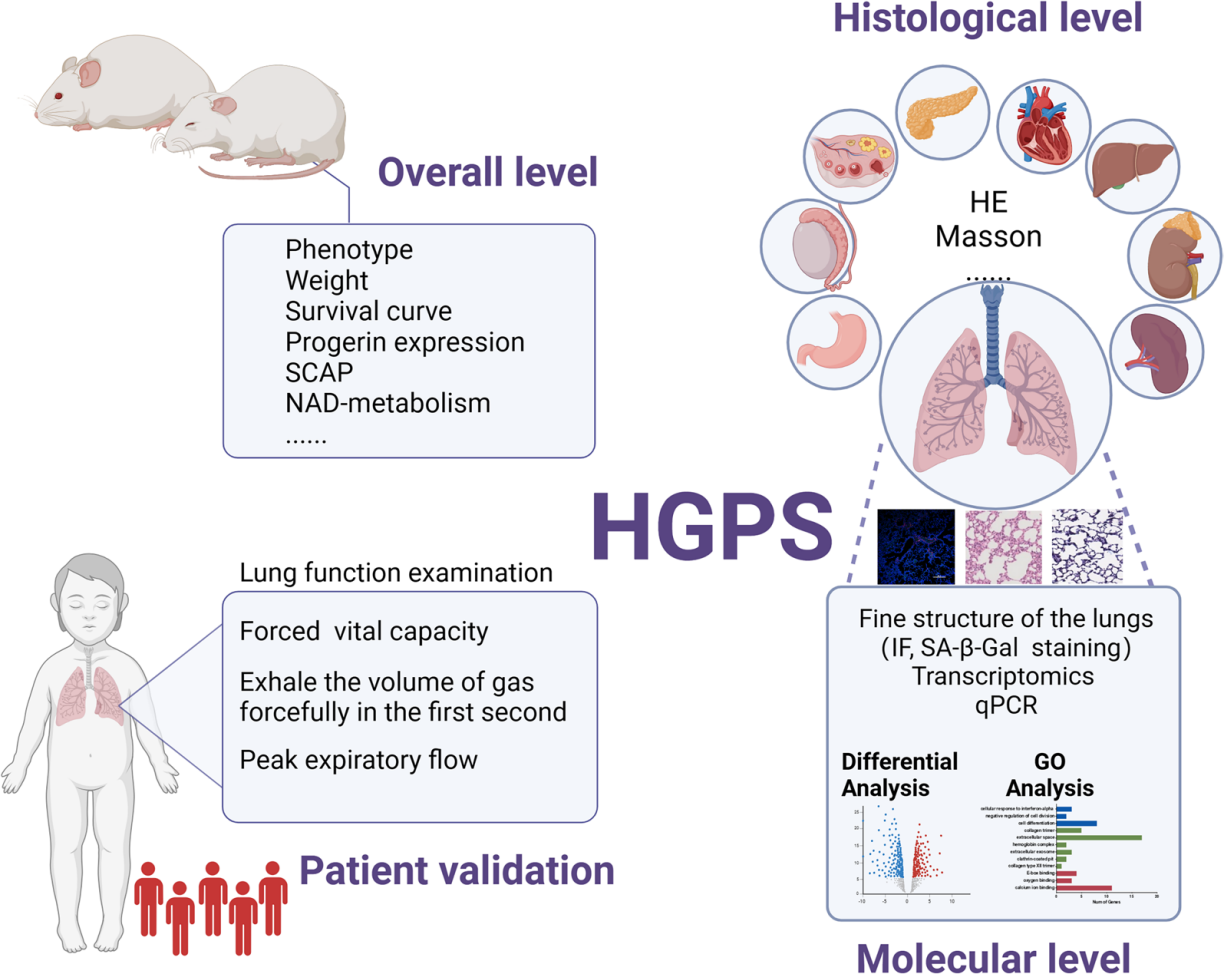

**Supplementary Information:**

The online version contains supplementary material available at 10.1186/s10020-025-01165-x.

## Introduction

Hutchinson–Gilford progeria syndrome (HGPS), or progeria, is a rare genetic disorder characterized by accelerated aging, impaired growth, disrupted lipid metabolism, and bone fragility. It leads to a significantly reduced lifespan, with an average expectancy of 13.5 years (Merideth et al. 2008; Osorio et al. 2011; Sinha et al. 2014). HGPS features manifest in multiple organs, including the skin and liver (Rork et al. 2014; Mahdi et al. 2020). Over 90% of HGPS cases result from a single point mutation (p.G608G) in exon 11 of the *LMNA* (*Lamin A/C*) gene (Piekarowicz et al. 2019; Koblan et al. 2021), which activates a cryptic splicing site and forms Progerin (Loi et al. 2016; Pablo Mayoral and López-Otín , 2018). Progerin may disrupt nuclear structural integrity, ultimately driving premature senescence and cell death (Pacheco et al. 2014; Danielsson et al. 2022). The scarcity of patient samples poses a challenge for in-depth investigations into HGPS pathophysiology mechanisms and clinical trials (Coppede 2013), hindering the development of novel therapies. In this challenging context, mouse models emerge as crucial tools, providing valuable insights into disease mechanisms and facilitating drug screening (Li et al. 2021).

Over the past few decades, several HGPS mouse models have been developed (Osorio et al. 2011; Pendas et al. 2002; Yang et al. 2005; Varga et al. 2006; Sagelius et al. 2008; Wang et al. 2008; Rosa et al. 2013). Notably, the G608G models, characterized by a G608G point mutation in the *LMNA* gene, are among the most utilized. These models accurately replicate key HGPS features, including arterial collagen fibril accumulation and the loss of vascular smooth muscle cells (Varga et al. 2006; Pablo Mayoral and López-Otín 2018). While previous reports have highlighted pathological changes only in arteries, skin, and cardiac function in HGPS mice, a comprehensive assessment of organ-specific alterations in other vital organs has been lacking. Therefore, our objective was to attain a more comprehensive understanding of the reliability of mouse models beyond the well-established cardiac and cutaneous manifestations, aiming to unravel the precise molecular mechanisms underlying HGPS.

In this study, we investigated the involvement of the lungs in HGPS pathogenesis, expanding on the well-established systemic manifestations of the disease. We initially employed the C57BL/6-Tg (*LMNA*^G608G^) HClns/J mouse model, carrying the G608G mutation, and observed substantial pathological changes in multiple organs, with a particular focus on the lungs. Additionally, we investigated the NAD metabolism pathway and changes in aging-related factors to validate the presence of accelerated aging. Subsequently, our transcriptomic analyses of lung tissues from HGPS mice revealed dysregulated pathways associated with lung functioning. To corroborate our findings from the mouse model, we conducted the first-ever cohort study of HGPS patients in China. Pulmonary function assessments indicated impaired pulmonary ventilation functions, including significantly reduced forced vital capacity, exhalation volume, and peak expiratory flow in HGPS patients compared to healthy individuals. These findings suggest that lung involvement may be an underrecognized aspect of HGPS pathogenesis. While our study provides preliminary evidence linking lung abnormalities to HGPS, further studies with larger cohorts and functional validation are necessary to confirm the potential role of pulmonary assessments in disease monitoring or therapeutic strategies (Graphical abstract).

## Materials and methods

### Animal experiments and organ collection

All experiments were conducted following protocols approved by The Animal Ethics Committee of Zhejiang University. Wild-type (WT) C57BL/6 mice (8 weeks) were purchased from Beijing Vital River Laboratory Animal Technology Co., Ltd. (Beijing, China). F0 heterozygous female and male C57BL/6-Tg (*Lmna*^G608G^) HClns/J mice were purchased from the Jackson Laboratory (Hemizygous for Tg (*Lmna*^G608G^) HClns, 010667). Homozygous and heterozygous mice were obtained by mating homozygous males with heterozygous females. We selected F2 homozygous male mice (*Lmna* ^G608G/G608G^) for subsequent experiments (n = 6–10). Organs (lung, heart, skin, aorta, testis, kidney, thymus, stomach and spleen) from 5-month-old mice were used in this study. Each experiment was independently repeated at least three times.

### Tissue paraffin histology

The lung, heart, skin, aorta, and other tissues were fixed in 4% paraformaldehyde (PFA) for 24 h, followed by dehydration using graded alcohols and embedding in paraffin. Cross-sections with a thickness of 4–5 μm were prepared and mounted on charged slides for visualization using H&E staining (Solarbio, China). Additional staining was performed using Masson's trichrome (Solarbio, China). Images were acquired using the Aperio ImageScope 12.4.6 imaging system at magnifications of 5X and 20X and analyzed using Image software. Subsequently, further processing of the images was performed using Adobe Illustrator 2022 software to achieve high-quality and clear visuals.

### SA-β-gal assay

Senescence-associated β-galactosidase (SA-β-Gal) activity was measured by a β-galactosidase staining kit (Beyotime, China) according to the manufacturer's instructions. Frozen sections with a thickness of 8–10 μm were prepared and mounted on charged slides for visualization using staining. Briefly, frozen sections were first rewarmed, and the tissues (lung, heart, skin, aorta, etc.) were washed 3 times with PBS for no less than 5 min each time. The remaining tissues were fixed by staining with fixation solution for 15 min at room temperature and then incubated at 37 °C with freshly prepared lysosome β-galactosidase substrate 5-bromo-4-chloro-3-indolyl β-D-galactopyranoside (X-Gal) solution (pH 6) overnight. Stained tissues were observed with an inverted microscope (Olympus, Japan), and images were recorded and compared with those obtained from WT mice.

### Immunofluorescence

The mouse tissues were dissected and placed onto the inner surface of an embedding mold coated with OCT, frozen sections with a thickness of 8–10 μm adhered to anti-slip slides. A solution containing 4% PFA was applied on top of the slices for fixation for 30 min. PBS was used to wash the tissues 3 times, each time lasting for 5 min. Droplets of 0.5% Triton X-100 were directly added above the tissues to facilitate permeation at RT for 20 min. Sealed with a solution containing 5% BSA at RT for about 1 h. Afterward, Progerin primary antibody was diluted in a solution containing 5% BSA and incubated overnight in a humidified chamber set at 4℃. The slices were rewarmed for half an hour and washed 3 times with PBS for 5 min each time. Fluorescent secondary antibodies corresponding to the species of primary antibodies were diluted in a solution containing 5% BSA to stain different target proteins within the tissues, and incubated without light exposure at RT for 2 h. Finally, PBS was used again to wash the specimens 3 times, each time lasting 10 min per wash cycle. DAPI anti-fluorescence quenching sealing tablets were applied directly above each specimen and allowed to dry naturally at RT for 1 h before observation under a confocal microscope. The images were analyzed using ImageJ software for co-localization analysis of Progerin and the certain lung major compartments marker. For each experimental condition, 10 random fields of view were selected to ensure unbiased sampling. The Pearson’s correlation coefficient (R value) was calculated to evaluate the degree of co-localization between the two proteins. Statistical significance was determined using ANOVA for analysis, with a *p* < 0.05 considered statistically significant.

### Western blot

The samples were rinsed twice with 1 × PBS and suspended in a 5 × SDS loading buffer. Frozen tissues (50 mg per sample) were homogenized in 300 mL of 100 mM Tris–HCl (pH 7.4), 2% SDS, and 50 mM EDTA using a Polytron homogenizer. Protein concentration was determined using the bicinchoninic acid (BCA) technique with the Pierce BCA Protein Assay Kit. Equal amounts of proteins were loaded onto 10% SDS–polyacrylamide gels. Following electrophoresis, the gels were transferred onto PVDF membranes. The membranes were then blocked with 5% nonfat dry milk in PBS-T buffer [140 mM NaCl, 10 mM phosphate buffer, and 0.05% Tween 20] in PBS for 1 h at room temperature. Subsequently, the membranes were incubated overnight at 4 °C or 1 h at room temperature with various primary antibodies, including monoclonal anti-Progerin (Santa Cruz, USA) at 1:500 and anti-actin (Yeasen Biotech, China) at 1:40,000. Subsequently, the blots were incubated with 1:10,000 goat anti-mouse horseradish peroxidase (HRP) (Jackson Immuno Research Laboratories, USA) in 1.5% nonfat milk in PBS-T. The blots were then washed, and the immunoreactive bands were developed using Immobilon Western chemiluminescent HRP substrate (Millipore, USA).

### Qualification of NAD metabolites determined by UHPLC-MS/MS

The dried metabolized extract was reconstituted in 100 μl 70% acetonitrile solution. An ultra-performance liquid chromatography-tandem mass spectrometry (UHPLC-MS/MS) method was developed for differential metabolite analysis. UHPLC-MS/MS system with the SCIEX QTRAP 6500 triple quadrupole mass spectrometer (SCIEX, USA), coupled to Nexera X2 LC-30AD detector (Shimadzu Corporation, Japan). This system utilized an Electrospray ionization (ESI) interface and a cooling autosampler. Operating conditions for the ESI were set as follows: temperature at 300 ℃, curtain gas at 30 psi, nebulizer gas, ion spray voltage (IS) at 5500 V/−4500 V, and spray gas and auxiliary heating gas at 35 psi, respectively.

Each analyte was performed in multi-reaction monitoring (MRM) parameters to determine the optimal conditions by fluidizing a single reference substance into an ESI source in either positive or negative ion mode. A gradient (0.5, 1.0, 2.0, 5.0, 10.0, 20.0, 50.0, 100, 500, 1000 ng/mL) of NAD, NAM, NNO, NA, NADH, 2-Py, MNA was applied simultaneously to the temperatures of vaporizing. Sensitivity and specificity were optimized, and parent and daughter ions specific to each molecule, capillary voltages, and collision energies. Peak areas were used for calculations by Tracefinder 3.2 (Thermo Fisher Scientific, USA).

### Total RNA isolation and RT‒PCR

Total RNA was extracted from certain tissues using TRIzol reagent (Thermo Fisher Scientific, USA) according to the instructions and purified with chloroform according to the manufacturer's protocol. The concentration of RNA was quantified using a NanoDrop spectrophotometer 1000 (Thermo Fisher Scientific, USA). Total RNA (0.5 mg) was used for reverse transcription, and a quantitative real-time qPCR assay was performed using SYBR Green (Vazyme, China). The primers for the targeted genes (*Thy1, Tnc, Col12a1, Cspg4, Eno2, Pfkp, Anpep, Prps1, p16, p27, Ki67, Il-6, Il-8, Vegf-a, Vegf-c, Mmp2, Ccr1, Slc2a3*) are shown in Table S1. The relative abundance of mRNA was obtained by normalization to actin levels.

### Transcriptomic profiling

20 mg of lung tissue collected from both HGPS and control mice was homogenized, mixed, and subsequently centrifuged for sample preparation. Total RNA was extracted from the samples using TRIzol Reagent (Life Technologies, USA). RNA concentration and purity were assessed using a NanoDrop 2000 (Thermo Fisher Scientific, USA). Subsequently, sequencing libraries were prepared using the Hieff NGS Ultima Dual-mode mRNA Library Prep Kit for Illumina (Yeasen Biotech, China) following the manufacturer's instructions, with unique index codes assigned to differentiate sequences from each sample. The libraries were then sequenced on an Illumina NovaSeq platform, and the raw data were processed using the BMKCloud (www.biocloud.net) online platform. Differential expression analysis was carried out using DESeq2. The resulting *p*-values were adjusted using the Benjamini and Hochberg method. Genes with an adjusted *p* < 0.01 and a fold change of ≥ 2 as determined by DESeq2 were considered as differentially expressed. Additionally, Gene Ontology (http://geneontology.org/docs/go-enrichment-analysis/) and the Kyoto Encyclopedia of Genes and Genomes (KEGG) (http://www.genome.jp/kegg/) pathway enrichment analyses were conducted to assess the statistical enrichment of differentially expressed genes.

### Pulmonary function assessment on HGPS patients

This research was performed in compliance with the ethical standards set by the Children's Hospital at Zhejiang University School of Medicine and was approved by the Ethics Committee (Ethical Protocol Number: 2023-IRB-0186-P-01). The study strictly adhered to the principles delineated in the Declaration of Helsinki, and the reporting of the research followed the comprehensive EQUATOR guidelines (Simera et al. 2010). Patients were enrolled according to strict pre-defined inclusion criteria. The most important inclusion criteria were as follows: age ≥ 1 year, classical or non-classical HGPS through genetic testing (*LMNA* or *ZMPSTE24* mutation). Patients carrying the classical *LMNA* G608G defined as classical HGPS, while other genes involved in nuclear envelope stability, such as *LMNA* (non-G608G variants) or *ZMPSTE24* (Zinc Metallopeptidase STE24), leading to a progeroid phenotype defined as HGPS-like PL (Progeroid Laminopathy). 6 participants were enrolled in a cohort, with three individuals diagnosed with HGPS and three diagnosed with HGPS-like PL patients. The average age within this cohort was 138.33 ± 41.18 months. Comprehensive pulmonary function assessments were conducted, involving measurements of forced vital capacity (FVC), forced expiratory volume in the first second (FEV1), and peak expiratory flow (PEF). Subsequently, the Tiffeneau-Pinelli index (FEV1/FVC ratio) was calculated. Written informed consents were obtained from all participants.

### Statistical analysis

Statistical comparisons among the different groups were made using Student’s *t*-test and ANOVA in GraphPad Prism (Version 8.0, GraphPad Software). We performed normality tests (such as the Shapiro–Wilk test or Kolmogorov–Smirnov test) on the data to assess whether the distributions followed a normal curve. Based on the results of these tests, we determined that parametric tests were appropriate for our data. *p* < 0.05 were considered statistically significant.

## Results

### HGPS mice are characterized by weight loss and a short lifespan

**Fig. 1:**
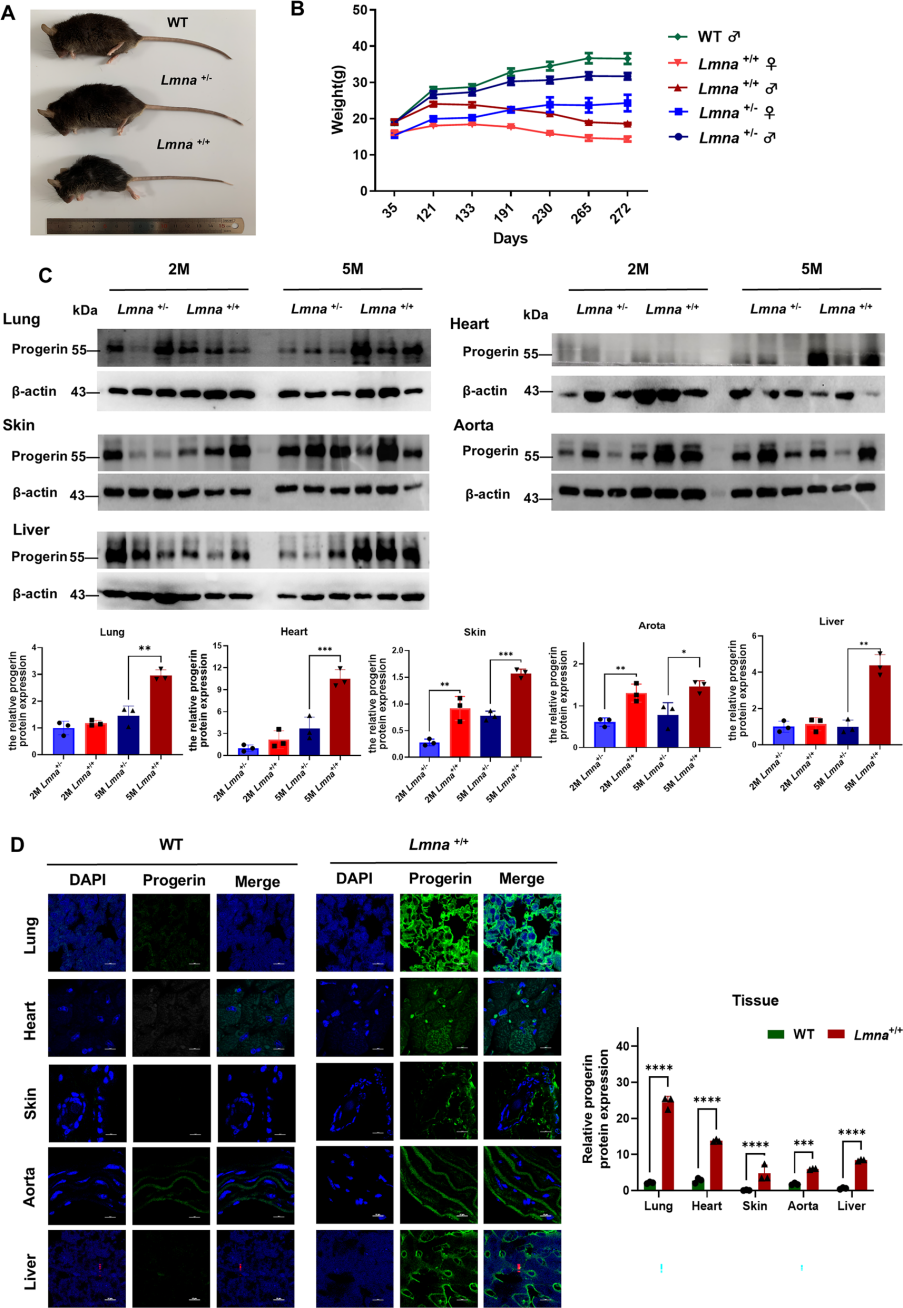
Phenotype and expression of Progerin in the C57BL/6-Tg (*Lmna*^*G608G*^) HClns/J mouse model. **A** Photographs of mice with homozygous (+ / +), wild-type (−/−), and heterozygous ( ±) genotypes. **B** Cumulative graph showing the body weight over time for wild-type (−/−), homozygous (+ / +), and heterozygous ( ±) mice. **C** Western blot analysis of Progerin and β-actin in the lung, heart, skin, aorta, and liver an of heterozygous (*Lmna*^G608G/+^) and homozygous (*Lmna*^G608G/G608G^) mice measured at 5 months. Quantitative analysis of the relative level of Progerin expression in these tissues of mice at 5 months. **D** Immunofluorescence analysis of frozen sections discloses the expression of Progerin in the lung, heart, skin, aorta, spleen, and kidney of wild-type (WT) and homozygous (*Lmna*.^G608G/G608G^) mice at 5 months. blue: DAPI, green: Progerin. (using Ordinary one-way ANOVA for analysis, ** stands for *p* < 0.01, *** stands for *p* < 0.001, **** stands for *p* < 0.0001)

Both homozygous (*Lmna*^G608G/G608G^, HGPS) and heterozygous (*Lmna*^G608G/+^) C57BL/6-Tg (*Lmna*^G608G^) HClns/J mouse models were utilized and compared to wild-type (WT) (Fig. S1A). At 5 months, homozygous HGPS mice exhibited a noticeably smaller body size than heterozygous individuals (Fig. 1A). We conducted periodic weight measurements and observed that homozygous mice, both females and males, experienced significant weight loss after reaching 4 months of age (Fig. 1B). Furthermore, the HGPS mice displayed a significantly reduced lifespan of 287 ± 3 days (Fig. S1B). These findings confirmed that the HGPS mice exhibited common phenotypes observed in HGPS patients.

### Progerin contents and aging degree in various tissues

Progerin, the pivotal hallmark of HGPS, was meticulously quantified and studied in various organs, including the lung, heart, skin, aorta, etc. We assessed Progerin levels in these organs at 5 months (Fig. S1C), with the lungs exhibiting the highest level of expression indicated by WB in HGPS mice, surpassing even the heart. As the manifestations of premature aging advanced, a significant increase in the Progerin expression was observed. At 5 months, HGPS mice consistently displayed higher Progerin levels than heterozygous by WB (Fig. 1C, S1D). A comparative analysis of the frozen sections of these organs following immunofluorescence staining revealed that the expression of Progerin was most pronounced in the lungs and heart (Fig. 1D, S1E). This intriguing finding suggests that, apart from the heart, as reported in previous studies, the lungs may be more significantly affected in HGPS than previously recognized, suggesting a need for further investigation into their potential contribution to disease progression.

### HGPS mice are characterized by abnormal NAD metabolism, cellular senescence and SASP signaling

**Fig. 2:**
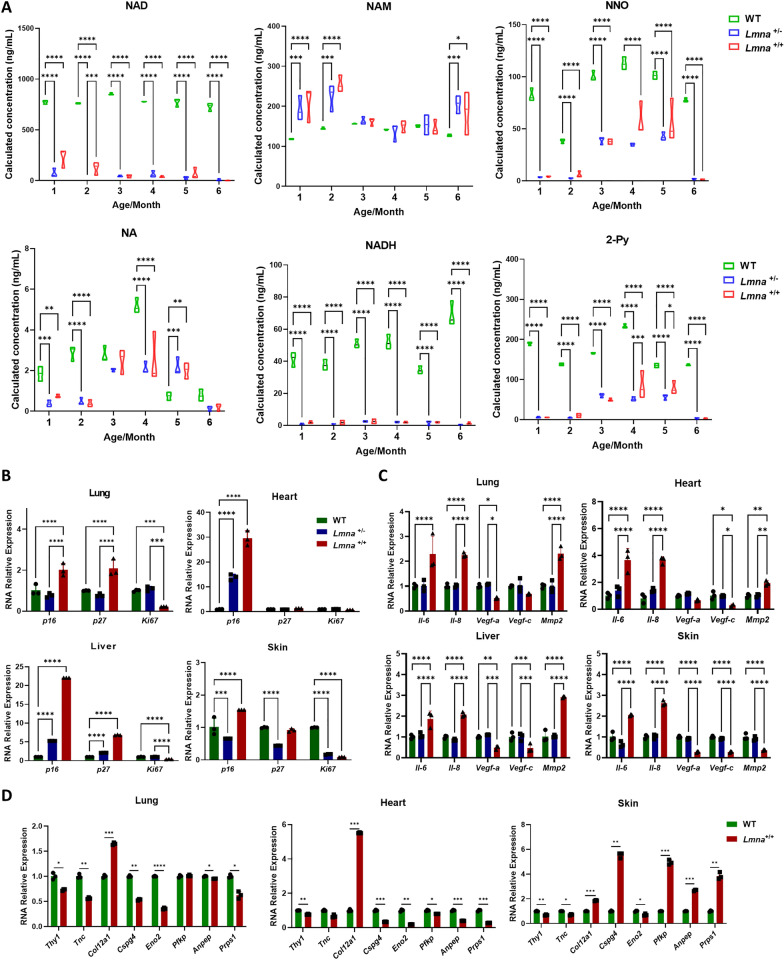
HGPS mice are characterized by abnormal NAD metabolism, cellular senescence and SASP signaling. **A** Calculated concentrations of NAD, NAM, NNO, NA, NADH, and 2-Py in heterozygous (*Lmna*^G608G/+^) and homozygous (*Lmna*.^G608G/G608G^) mice measured from month 1 to month 6 compared to the control group. NAD = nicotinamide adenine dinucleotide; NAM = N′-methylnicotinamide; NNO = nicotinamide-N-oxide; NA = Nicotinic acid; NADH = Nicotinamide adenine dinucleotide; 2-Py = N′-methyl-2-pyridone-5-carboxamide. **B** Levels of relative RNA expression of *p16, p27,* and *Ki67* in mice's lung, heart, liver, and skin with different genotypes measured at 2 months and 5 months. **C** Levels of relative RNA expression of *Il-6, Il-8, Vegf-a, Vegf-c, and Mmp2* in mice's lung, heart, liver, and skin with different genotypes measured at 2 months and 5 months. **D** Analysis of the expression levels of aging-associated genes using qPCR. (using Ordinary one-way ANOVA for analysis, ** stands for *p* < 0.01, *** stands for *p* < 0.001, **** stands for *p* < 0.0001)

NAD and its associated metabolites play pivotal roles in maintaining physiological processes and are closely linked to natural aging (Yaku et al. 2018). In our study, we monitored six metabolites involved in NAD metabolism for six consecutive months in serum: nicotinamide adenine dinucleotide (NAD), nicotinamide (NAM), nicotinamide-N-oxide (NNO), nicotinic acid (NA), Nicotinamide adenine dinucleotide (NADH), and N-methyl-2-pyridone-5-carboxamide (2-Py). We observed a significant reduction level in NAD + , NNO, NA, NADH, and 2-Py in HGPS mice compared to the WT group (Fig. 2A), suggesting that HGPS and natural aging may have similar NAD network associations. Moreover, we conducted quantitative PCR (qPCR) to assess the expression of three well-known biomarkers associated with cellular senescence markers, namely, *p16, p27*, and *Ki67*, across multiple organs. As anticipated, while demonstrating age-dependent variations, the HGPS mice universally exhibited significantly higher levels of *p16* and *p27* than the WT mice across all organs (Fig. 2B). In contrast, *Ki67* exhibited significantly lower levels in the HGPS mice. Moreover, we measured the level of senescence-associated secretory phenotype (SASP), such as IL-6 (Interleukin 6), IL-8 (Interleukin 8), VEGF-a (Vascular endothelial growth factor a), VEGF-c (Vascular endothelial growth factor c), and MMP2 (matrix metallopeptidase 2), the HGPS mice universally exhibited significantly higher levels of *Il-6, Il-8* and *Mmp2* than the WT mice across all organs (Fig. 2C). In contrast, *Vegf-a* and *Vegf-c* exhibited significantly lower levels in the HGPS mice. In a literature review focused on human cell lines, genes reported to exhibit abnormal expression in HGPS were examined (Mateos et al. 2018a). It was identified that the *Thy1 (Thy-1 cell surface antigen)* and *Tnc (tenascin C)* gene expression in the HGPS group were significantly lower compared to the WT group among heart, lung, and skin tissues (Fig. 2D). It is well-known that genetic ablation of CSPG4-expressing (Chondroitin sulfate proteoglycan 4) cells resulted in excessive vascular permeability, a decline in cardiac function and increased mortality (Quijada et al. 2023), and the relative lower expression of CSPG4 was validated both in lung and heart.

### Histological analyses reveal anomalies in multiple organs, especially for lung

**Fig. 3:**
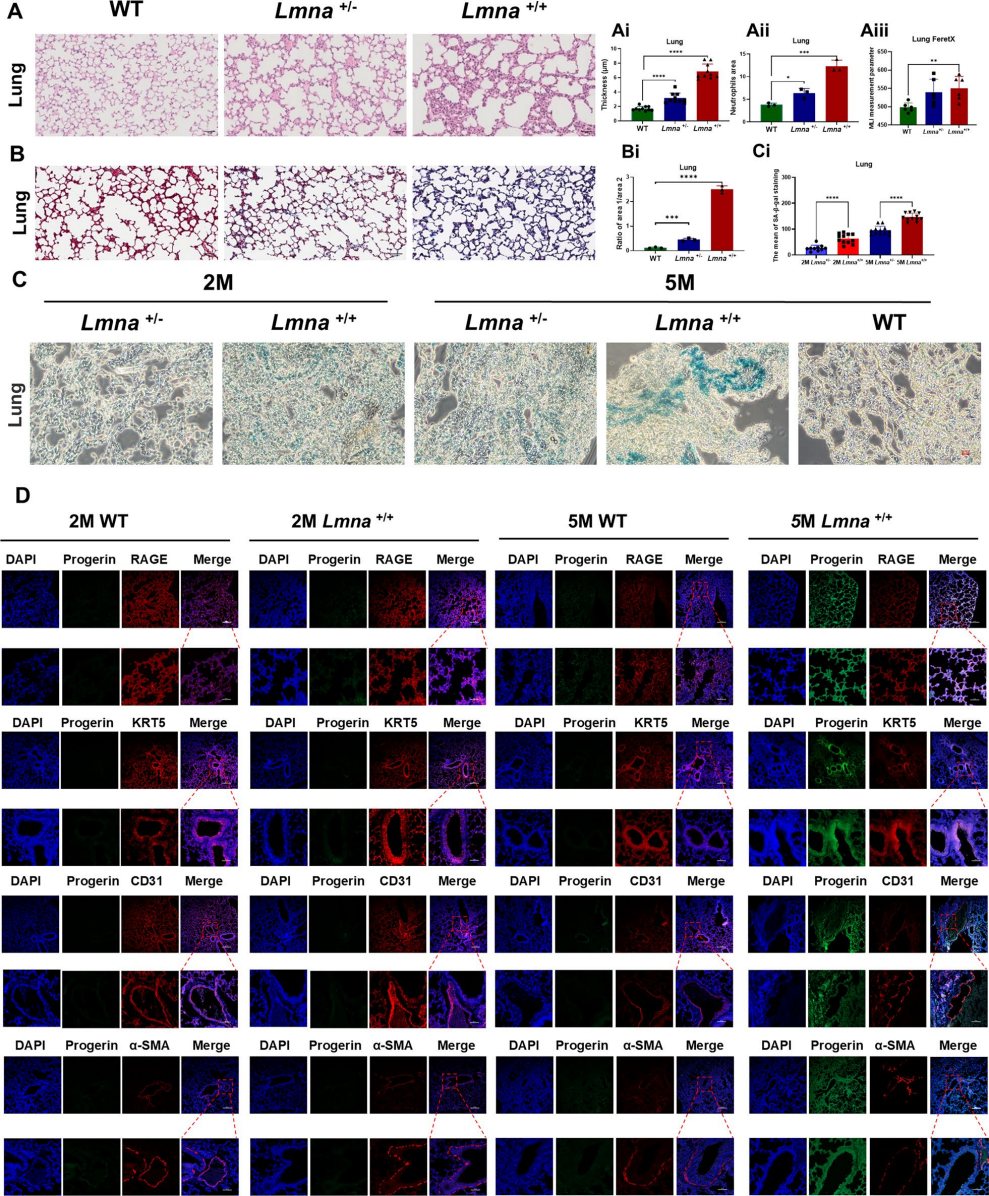
Histological analyses reveal anomalies in lung tissue. **A** Paraffin section of lung tissue stained with hematoxylin and eosin (HE) reagents. Measurement of lung tissue thickness (Ai), area of neutrophils (Aii), and the mean linear intercept (MLI) value (Aiii) in mice with different genotypes. **B** Paraffin section of lung tissue stained with Masson trichrome reagents. Measurement of pulmonary fibrosis (Bi) in mice with different genotypes. **C** β-galactosidase (SA-β-Gal) staining of frozen sections in the lung tissue at 2 and 5 months. (using Ordinary one-way ANOVA for analysis, ** stands for *p* < 0.01, *** stands for* p* < 0.001, **** stands for *p* < 0.0001). **D** Immunofluorescence analysis of frozen sections disclose the expression of Progerin in the lung of wild-type (−/−) and homozygous (+ / +) mice at 5 months. blue: DAPI, green: Progerin, red: the certain lung major compartments marker

Paraffin sections and frozen sections were obtained from various organs and subjected to hematoxylin and eosin (H&E) staining and Masson's trichrome. As HGPS progressed, the structure of the alveolar septa gradually diminished, lung cells dispersed throughout the lung tissue, the alveolar wall thickening, the area of inflammatory cell infiltration increased, and mean linear intercept length Lm enlarger (Fig. 3A, Ai, Bii). Meanwhile, Masson's trichrome staining indicated lung tissue of HGPS have significant fibrosis (Fig. 3B, Bi). Picro-sirius red staining was used for the analysis of cardiac fibrosis. The more red or purple areas and the denser they are (the greater the ratio of the red area divided by the blue area), the more severe the degree of fibrosis. The HGPS mice demonstrated notable impairment in the cardiovascular system. Significant collagen accumulation indicating severe myocardial fibrosis was observed in the heart tissues, as shown by the increased red area under Picro-Sirius Red staining (Figs. S2A, S2Ai). Furthermore, the skin tissue exhibited reduced thickness of the dermal layer and accumulation of melanin in HGPS mice compared to WT mice (Figs. S2B, S2Bi, S2Bii). In the aorta tissue, the HGPS mice demonstrated a decreased thickness of the tunica media, possibly caused by the loss of VSMCs, along with an increased thickness of the tunica adventitia, possibly attributed to vascular stiffening (Figs. S2C, S2Ci, S2Cii). These findings were consistent with previous studies (Osorio et al. 2011; Murtada et al. 2020). The staining of the liver tissue was performed in regions around the hepatic artery and revealed decreased hepatocyte volume and a noticeable shrinkage of cell nuclei, resulting in an increased nuclear-cytoplasmic ratio in HGPS mice (Figs. S2D, S2Di). Moreover, the HGPS group showed a substantial reduction in the germinal center area of the spleen, with red pulp infiltrating white pulp, causing indistinction on the boundaries (Fig. S2E, S2Ei). The kidney tissue exhibited a significant reduction in the number of glomerular cells, disorders of renal tubular cells and reduced cell number in the kidneys compared to WT or heterozygous mice (Fig. S2F, S2Fi, S2Fii). It is worth noticing that heart, liver, and kidney tissues universally demonstrated significant fibrosis, as indicated by Masson's trichrome staining (Figs. S3A, S3B, S3D, S3Ai, S3Bi, S3Di). Furthermore, the ovarian tissue in the HGPS group demonstrated a notable decrease in the quantity of mature follicles compared to the WT mice (Figs. S3C, S3Ci).

The lung tissue frozen sections were additionally subjected to senescence-related β-galactosidase staining. At 5 months, HGPS mice displayed widespread aging changes, notably in the lungs, as indicated by darker blue staining (Fig. 3C, Ci). To further clarify the affected regions, we also co-stained for Progerin accumulation, which allowed us to correlate Progerin expression with specific lung compartments. Based on our current results, Progerin shows strong colocalization with RAGE (Advanced glycosylation end-product specific receptor) and KRT5 (Keratin 5), suggesting that these compartments may be affected in HGPS lung pathology (Fig. 3D, S3F).

### Transcriptomic profiling of lung unveiled altered gene expression associated with aging pathways

**Fig. 4:**
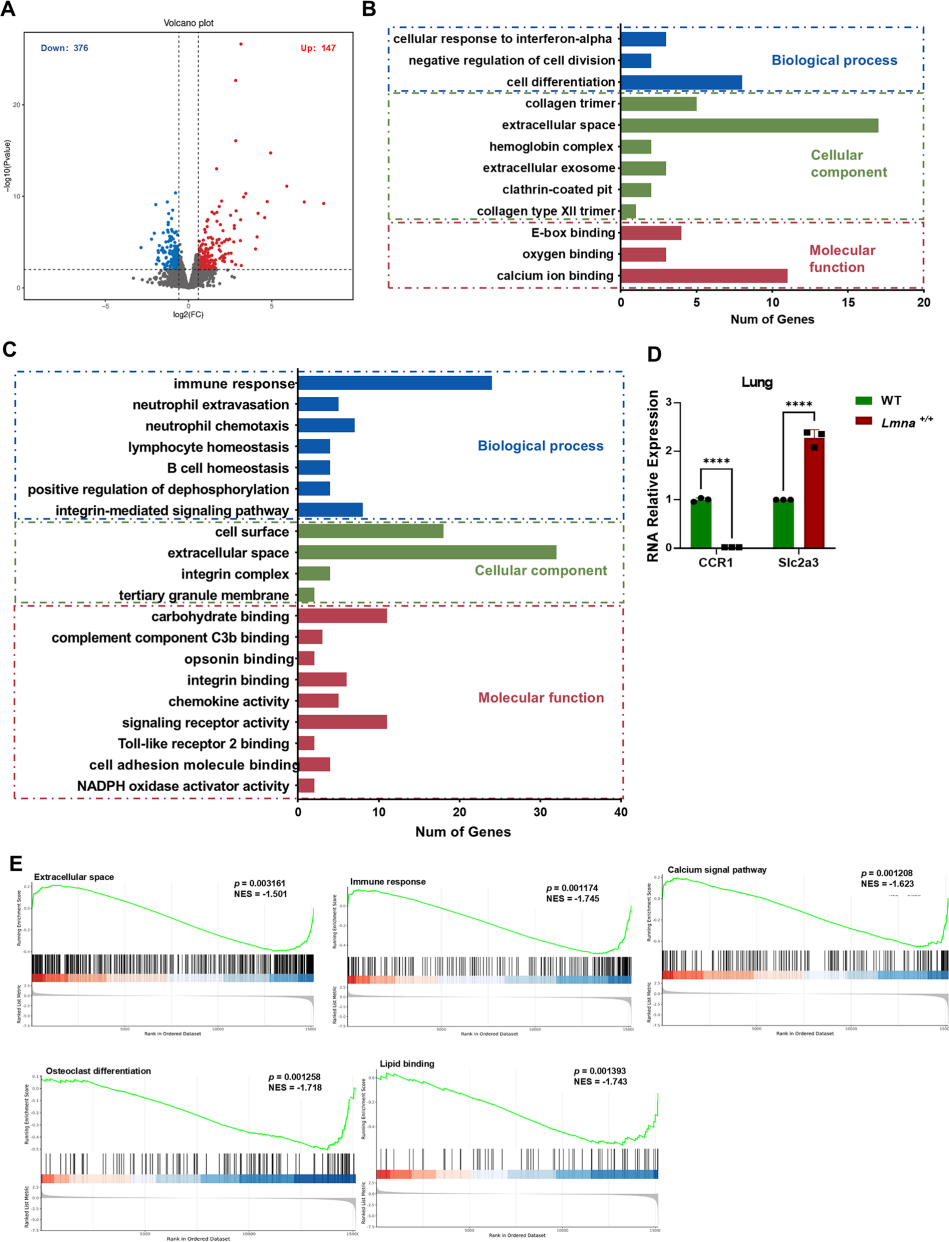
Transcriptomic analysis unveiled the differential expression of lung tissue-related genes. **A** Volcano map of transcriptome genes. Red dots, upregulated genes; blue dots, downregulated genes; gray dots, genes with no significance. **B** GO enrichment analysis of up-regulated DEGs. The DEGs were categorized into three main categories, Molecular Functions (MF), Biological Processes (BP), and Cellular Components (CC). **C** GO enrichment analysis of down-regulated DEGs. Enrichment pathway analysis of Cellular Components related genes. **D** qPCR validation the expression of typical differentiate genes. (using Ordinary one-way ANOVA for analysis, ** stands for *p* < 0.01, *** stands for *p* < 0.001, **** stands for *p* < 0.0001). **E** Gene Set Enrichment Analysis of pathways associated with extracellular space, immune response, lipid binding, Calcium signaling pathway and Osteoclast differentiation

To delve into the molecular mechanisms underlying observed lung alterations, we conducted transcriptomic on lung tissues from our HGPS mouse model and controls. As depicted in Fig. 4A, we identified 527 differentially expressed genes (DEGs), with 376 showing downregulation and 147 upregulation. Further GO analysis was conducted to delineate the significant functional DEGs in HGPS mouse lungs. As illustrated in Fig. 4B, up-regulated genes were mainly enriched in the processes associated with response to stimulus, cell differentiation, and apoptosis. Down-regulated genes were mainly enriched in the processes associated with immune response, vascular smooth muscle contraction, and circadian rhythm (Fig. 4C). *Ccr1(C–C motif chemokine receptor 1)* and *Slc2a3 (solute carrier family 2 member 3,* encoding Glucose Transporter 3, Glut3*)* were chosen because they were identified as significantly downregulated (*Ccr1*) and significantly upregulated (*Slc2a3*) in our transcriptomic analysis. Additionally, both genes have been reported to be associated with aging-related processes. CCR1 has been implicated in immune aging and inflammatory responses (Yung et al. 2007; Zhao et al. 2020). SLC2A3 plays a key role in glucose metabolism and neuronal function, and GLUT3 expression changes are associated with aging-related neurodegenerative diseases, including Alzheimer’s disease (Shin et al. 2018). The levels of *Ccr1* and *Slc2a3* were identified by qPCR, consistent with the transcriptomic analysis (Fig. 4D). Through GSEA analysis, we obtained results similar to those above, including extracellular space, lipid binding, calcium signaling pathway and osteoclast differentiation in aging (Fig. 4E).

### Pulmonary function assessment from patients validated lung involvements in HGPS

To confirm our findings in a clinical context, we conducted a cohort study that recruited children diagnosed with HGPS and assessed their pulmonary function. Based on clinical inclusion criteria, we enrolled six patients, including three with classical HGPS and three with non-classical HGPS. Table 1 provided detailed genotypic and phenotypic data for these patients. Pulmonary function assessments revealed substantial impairments in ventilation among HGPS patients, as indicated by significant reductions in forced vital capacity (FVC), exhalation volume (FEV1), and peak expiratory flow (PEF) compared to healthy controls (Table 2). These findings suggest that pulmonary dysfunction is a prominent feature of HGPS, HGPS-like or Atypical Progeria Syndrome, further supporting our observations from the mouse model and highlighting the need for closer respiratory monitoring in HGPS patients.

**Table 1:** Genotype and phenotype in Chinese patients with progeria and progeria-like lamin

No	Gender	Age	Clinical symptoms pulmonary ventilation	First symptoms	Age at first start						Genetic report
					Walk independently	Joint stiffness	Headache	Chest tightness	Cerebral infarction	Myocardial infarction	
1	Male	11y10m	Moderately abnormal	Hard, swollen skin	18 m	1y3m	/	/	/	/	LMNA c.1824 (exon 11) C > T Heterozygous mutations
2	Male	9y5m	Normal	Hard, swollen skin	15 m	1y3m	4y	/	/	10y	LMNA c.1824 (exon 11) C > T Heterozygous mutations
3	Female	7y4m	Normal	Hard, swollen skin	14 m	2y	/	/	/	/	LMNA c.1824 (exon 11) C > T Heterozygous mutations
4	Female	17y3m	Severe abnormality	Growth retardation	18 m	1y3m	/	/	/	/	LMNA c.1579 (exon 9) C > T Homozygous mutations
5	Male	15y3m	Mild abnormalities	Skin pigmentation	17 m	3y1m	/	/	/	9y	LMNA c.1453_1454delins AG (exon 8) Heterozygous mutations
6	Male	8y1m	Abnormal, mild restrictive ventilatory dysfunction with small airway dysfunction	Hard, swollen skin; joint stiffness	16 m	1 m	/	/	/	6y	ZMPSTE24 c.743C > T & c.469C > T Complex Heterozygous mutations

**Table 2:** Lung function in Chinese patients with HGPS, HGPS-like (PL), or Atypical Progeria Syndrome (APS)

Variables	Total (6)	HGPS (3)	PL or APS (3)
Man, [%]	4 [66.7]	2 [66.7]	2 [66.7]
Age (month)	138.33 ± 41.18	114.33 ± 27.03	162.33 ± 57.84
FVC (L)	1.10 ± 0.78	0.76 ± 0.04	1.43 ± 1.09
FVC % predicted value	71.00 ± 21.27	81.70 ± 12.88	60.30 ± 24.93
FEV1 (L)	1.02 ± 0.72	0.70 ± 0.06	1.32 ± 0.99
FEV1% predicted value	75.18 ± 21.90	85.60 ± 11.84	64.77 ± 27.08
FEV1/FVC (%)	93.34 ± 4.00	92.23 ± 3.56	94.45 ± 4.85
FEV1/FVC % predicted value	103.13 ± 4.56	104.47 ± 0.76	101.80 ± 6.79
PEF (L/S)	3.09 ± 1.30	2.47 ± 0.73	3.71 ± 1.59
PEF % predicted value	96.37 ± 27.26	114.23 ± 20.41	78.5 ± 21.98
Abnormal pulmonary ventilation function	4 [66.7]	1 [33.3]	3 [100]

*FVC* forced vital capacity, *FEV1* Exhale the volume of gas forcefully in the first second, *PEF* Peak expiratory flow

## Discussion

In our study, *Lmna*^G608G/G608G^ mice showed features that include premature death, weight loss, and abnormal postures (Fig. 1 and S1), which is in line with previous studies on patients (Gordon et al. 1993; Kieran et al. 2007; Ullrich and Gordon 2015) as well as other animal models (Osorio et al. 2011; Kreienkamp et al. 2019; Zaghini et al. 2020). And in concurrence with prior cardiovascular anomalies in both animal models (Rivera-Torres et al. 2016; Kato and Maezawa 2022), our study revealed that multiple organs (lung, heart, skin, aorta, etc.) were compromised in the view of Progerin contents, histology, NAD metabolism, SASP signaling (Figs. 1, 2, and 3). Notably, HGPS and natural aging may have similar NAD networks, genes-associated SCAP expression profiling in our study (Fig. 2) (Braidy et al. 2011; Gomes et al. 2013; McReynolds et al. 2020).

Recently, Krüger et al*.* investigated multi-organ pathology in *Lmna*^G609G/G609G^ mice, a different model exhibiting severe fibrosis, inflammation, and vascular dysfunction across multiple organs (Kruger et al. 2024). While in our study, beside emphasized systemic degeneration, our study focuses on revealing significant lung abnormalities, including emphysema-like changes and alveolar structural alterations. The lungs displayed the highest Progerin expression among all organs in our HGPS mice at 5 months, because the C57BL/6-Tg (*Lmna*^G608G^) HClns/J model develops severe pathological phenotypes at this stage, including vascular defects, muscle atrophy, and systemic aging features (16) (Figs. 1 and 3). Initially, we observed enhanced lung fibrosis in HGPS mice, akin to idiopathic pulmonary fibrosis (IPF), a condition recognized for its age-related positive correlation (Gulati and Thannickal 2019), leading to dyspnea and progressive lung function decline (Blackwell et al. 2014; Renzoni et al. 2014; Selman and Pardo 2014). The HGPS mice exhibited mild intrapulmonary hemolysis and immune cell infiltration, significantly longer length of mean linear intercept values, resembling features seen in patients experiencing normal aging and HGPS patients (Schneider et al. 2021). Concurrently, heightened inflammation in the mouse lungs resembled a characteristic feature of chronic obstructive pulmonary disease (COPD), a typical ailment linked to aging (King 2015; Kubben and Misteli 2017; Faniyi et al. 2022). Intriguingly, our pioneering cohort study on HGPS patients also validated impaired lung ventilation function (Table 2). And these pulmonary manifestations were previously documented in a singular Iraqi HGPS case report (Faiq et al. 2019). Additionally, transcriptomic analyses revealed enrichment of genes related to the extracellular matrix and extracellular space, which are known to be associated with tissue fibrosis (Fig. 4B, C, E). KEGG analysis validated dysregulated pathways associated with cellular senescence and vascular smooth muscle contraction, which are key features in HGPS. Alterations in extracellular matrix (ECM)-receptor interactions were strongly associated with pulmonary fibrosis pathology (King 2015; Kreienkamp and Gonzalo 2020; Panyard et al. 2022). The upregulation of the glutathione system and the downregulation of the calcium signaling pathway are related to oxidative stress and inflammation, which are hallmarks of premature aging and natural aging, consistent with the research on mitochondrial dysfunction (Fig. 4) (Fafián-Labora et al. 2021; Mateos et al. 2018b; Tang et al. 2022). Our recent study showed that clearing abnormal mitochondria can improve the phenotype of HGPS mice and extend their lifespan (Sun et al. 2024). Partial alterations were lipid-related, consistent with previous findings indicating a significant decrease in fat stores in HGPS patients (Kreienkamp and Gonzalo 2020). In summary, our transcriptomic data further supported the resemblance between the HGPS mouse model and clinical patients, highlighting that lung abnormalities were a significant aspect in the diagnosis and treatment of HGPS diseases.

While our study provides valuable insights into lung-associated pathophysiology in HGPS, several limitations must be acknowledged. First, the sample size of both our animal model and human cohort is relatively small, which may limit the generalizability of our findings. Second, although histological and molecular analyses support our conclusions, functional validation through in vivo lung function assessments in mice and mechanistic studies on Progerin's effects in pulmonary cells are needed to confirm causality. Third, the human patient data were derived from a limited clinical cohort, and external validation using larger, multi-center datasets is required. Fourth, while classification into HGPS, HGPS-like and atypical progeria syndrome (APS) is based on specific *LMNA* or *ZMPSTE24* mutations, we were unable to directly quantify Progerin or farnesylated prelamin A levels in HGPS-like and APS patients due to limited availability of detection kits. Since Progerin/prelamin A accumulation is a key determinant of disease classification, this remains a gap between Progerin level with lung aging phenotype between our mouse model findings and human cases. Lastly, while our transcriptomic and biochemical analyses identified dysregulated pathways, further experimental validation is necessary to establish direct mechanistic links between Progerin accumulation, NAD metabolism, and lung pathology in HGPS.

In this study, we adopted a holistic approach, surpassing the confines of conventional HGPS research to explore interconnected pathways and assess multiple organs. Our findings provide additional evidence of systemic organ involvement in HGPS, with a particular focus on lung pathology. Notably, alterations in NAD metabolism observed in HGPS mice exhibit similarities to those seen in aging; however, further studies are needed to determine whether these changes directly contribute to disease progression. Furthermore, the lung abnormalities identified in HGPS mice closely parallel those found in patients, a correlation further substantiated by detailed omics data. Our findings emphasize the significance of pulmonary manifestations in HGPS and suggest that the disease affects multiple organ systems beyond those previously recognized. While our study contributes novel insights, additional research is required to validate these findings and determine whether lung-targeted interventions could be beneficial in HGPS management. HGPS shares various commonalities with the natural aging process, *e.g.,* multiple epigenetic changes, cardiac fibrosis, and Progerin accumulation (McClintock et al. 2007; Mosevitsky 2022; Benedicto et al. 2021). Therefore, studying HGPS pathogenesis also provides valuable insights into unveiling the intricate mechanisms underlying the natural aging process.

## Supplementary Information

Supplementary material 1.

## Data Availability

No datasets were generated or analysed during the current study.

## Abbreviations

2-Py: N’-methyl-2-pyridone-5-carboxamide
Anpep: Alanyl aminopeptidase
BCA: Bicinchoninic acid
BP: Biological processes
CC: Cellular components
Ccr1: C–c motif chemokine receptor 1
COPD: Chronic obstructive pulmonary disease
Col12a1: Collagen type xii alpha 1 chain
Cspg4: Chondroitin sulfate proteoglycan 4
ECM: Extracellular matrix
ESI: Electrospray ionization
FEV1: Forced expiratory volume in the first second
FVC: Forced vital capacity
H&E: Hematoxylin and eosin
Glut3: Glucose transporter 3
HGPS: Hutchinson–Gilford progeria syndrome
HRP: Horseradish peroxidase
Il-6: Interleukin 6
Il-8: Interleukin 8
KEGG: The Kyoto Encyclopedia of Genes and Genomes
Ki67: Marker of proliferation ki-67
Krt5: Keratin 5
Lmna/LMNA: Lamin A/C
MF: Molecular functions
Mmp2: Matrix metallopeptidase 2
MRM: Multi-reaction monitoring
NA: Nicotinic acid
NAD: Nicotinamide adenine dinucleotide
NADH: Nicotinamide adenine dinucleotide
NAM: N’-methylnicotinamide
NNO: Nicotinamide-N-oxide
p16: H3 histone pseudogene 10
p27: H3 histone pseudogene 23
PEF: Peak expiratory flow
PFA: Paraformaldehyde
Pfkp: Phosphofructokinase
Prps1: Phosphoribosyl pyrophosphate synthetase 1
qPCR: Quantitative PCR
Rage: Advanced glycosylation end-product specific receptor
SASP: Senescence-associated secretory phenotype
SA-β-Gal: Senescence-associated β-galactosidase
Slc2a3: Solute carrier family 2 member 3
Thy1: Thy-1 cell surface antigen
Tnc: Tenascin c
UHPLC-MS/MS: Ultra-performance liquid chromatography-tandem mass spectrometry
Vegf-a: Vascular endothelial growth factor a
Vegf-c: Vascular endothelial growth factor c
Zmpste24: Zinc metallopeptidase ste24
