# Supplementary material for: Disease pathogenicity in Hutchinson–Gilford progeria syndrome mice: insights from lung-associated alterations
Source: Mol Med. 2025 Mar 24;31:114. doi: 10.1186/s10020-025-01165-x (PMC11934591; doi:10.1186/s10020-025-01165-x)
Supplement: Supplementary file 1 — Supplementary material 1. [file 10020_2025_1165_MOESM1_ESM.docx]

**Table S1.** The primer sequences for qRT-PCR.

| Primer Name | Primer sequence (5’→3’) |
| --- | --- |
| *Thy1* | F: TGCTCTCAGTCTTGCAGGTG  R: TGGATGGAGTTATCCTTGGTGTT |
| *Tnc* | F: ACGGCTACCACAGAAGCTG  R: ATGGCTGTTGTTGCTATGGCA |
| *Col12a1* | F: AAGTTGACCCACCTTCCGAC  R: GGTCCACTGTTATTCTGTAACCC |
| *Cspg4* | F: GGGCTGTGCTGTCTGTTGA  R: TGATTCCCTTCAGGTAAGGCA |
| *Eno2* | F: AGGTGGATCTCTATACTGCCAAA  R: GTCCCCATCCCTTAGTTCCAG |
| *Pfkp* | F: GAAACATGAGGCGTTCTGTGT  R: CCCGGCACATTGTTGGAGA |
| *Anpep* | F: ACGCTCAGGAGAAGAATAGGAA  R: CTTAGGCAAGCGATACTGGTTC |
| *Prps1* | F: CCAAACATCAAACTCTTCAGCG  R: ACACTCTCACCAATTTCCACG |
| *p16* | F: TGTTGAGGCTAGAGAGGATCTTG  R: CGAATCTGCACCGTAGTTGAGC |
| *p27* | F: AGCAGTGTCCAGGGATGAGGAA  R: TTCTTGGGCGTCTGCTCCACAG |
| *Ki67* | F: GAGGAGAAACGCCAACCAAGAG  R: TTTGTCCTCGGTGGCGTTATCC |
| *Ccr1* | F: CTCATGCAGCATAGGAGGCTT  R: ACATGGCATCACCAAAAATCCA |
| *Slc2a3* | F: ATGGGGACAACGAAGGTGAC  R: GTCTCAGGTGCATTGATGACTC |
| *β-actin* | F: CATTGCTGACAGGATGCAGAAGG  R: TGCTGGAAGGTGGACAGTGAGG |
| *Il-6* | F: TACCACTTCACAAGTCGGAGGC  R: CTGCAAGTGCATCATCGTTGTTC |
| *Il-8* | F: GGTGATATTCGAGACCATTTACTG  R: GCCAACAGTAGCCTTCACCCAT |
| *Vegf-a* | F: CTGCTGTAACGATGAAGCCCTG  R: GCTGTAGGAAGCTCATCTCTCC |
| *Vegf-c* | F: CCTGAATCCTGGGAAATGTGCC  R: CGATTCGCACACGGTCTTCTGT |
| *Mmp2* | F: CAAGGATGGACTCCTGGCACAT  R: TACTCGCCATCAGCGTTCCCAT |
| *Gapdh* | F: ACCCTTAAGAGGGATGCTGC  R: CCCAATACGGCCAAATCCGT |


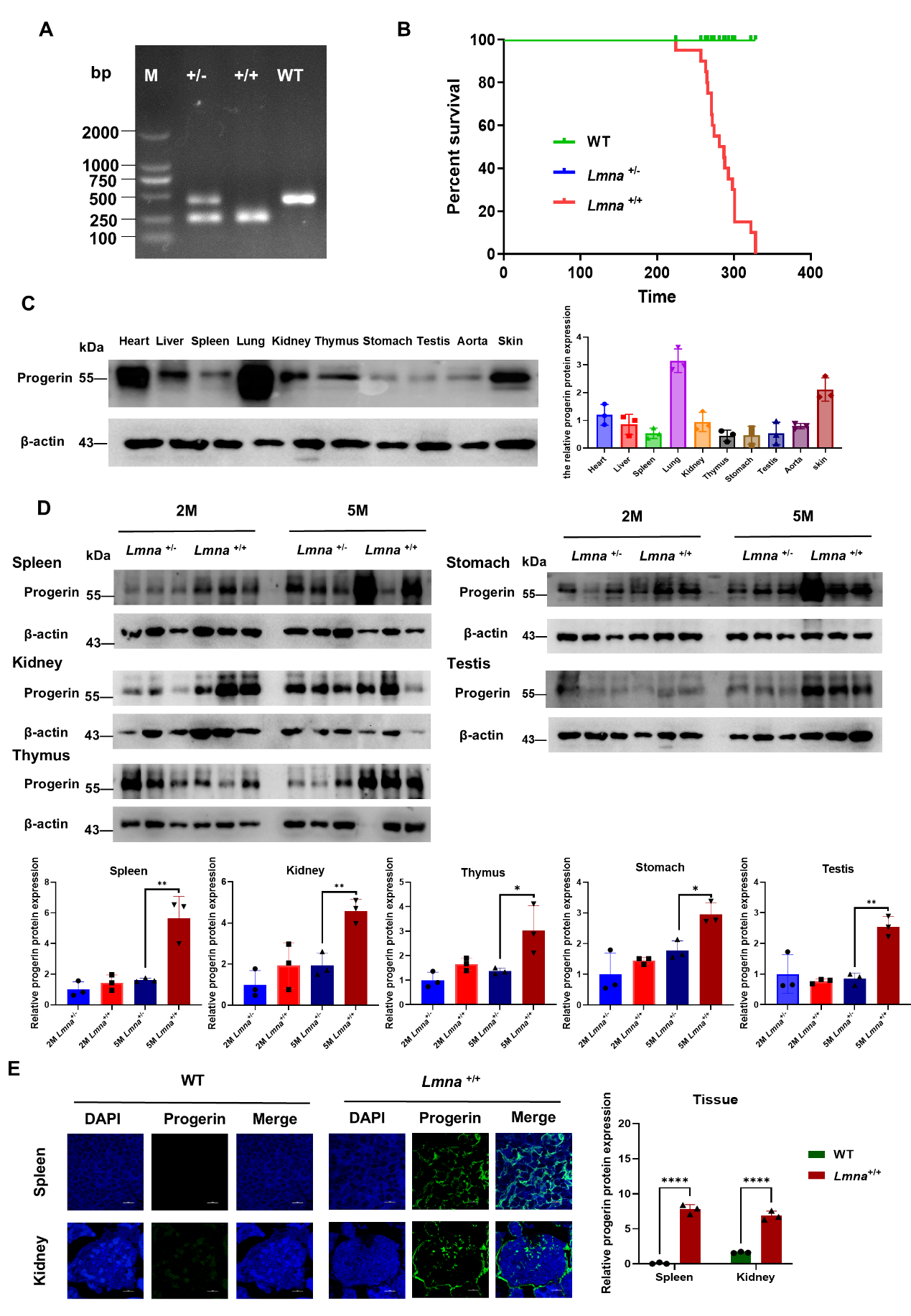


**Figure S1. Genotyping of C57BL/6-Tg (*Lmna**G608G) HClns/J mouse models.** (A) Independent genotyping of mice -/-, +/+, and +/- genotypes by PCR-based assay. (B) Survival plots of mice with different genotypes. (C) Quantitative analysis of the relative level of Progerin expression in the heart, liver, spleen, lung, kidney, thymus, stomach, and testis of mice at 5 months. (D) Western blot analysis of Progerin and actin in the spleen, kidney, thymus, stomach, and testis of heterozygous (+/-) and homozygous (+/+) mice measured at 2 months and 5 months. (E) Immunofluorescence analysis of frozen sections disclose the expression of Progerin in the spleen and kidney of wild-type (-/-) and homozygous (+/+) mice at 5 months. blue: DAPI, green: Progerin. (using Ordinary one-way ANOVA for analysis, ** stands for *p* < 0.01, *** stands for *p* < 0.001, **** stands for *p* < 0.0001).


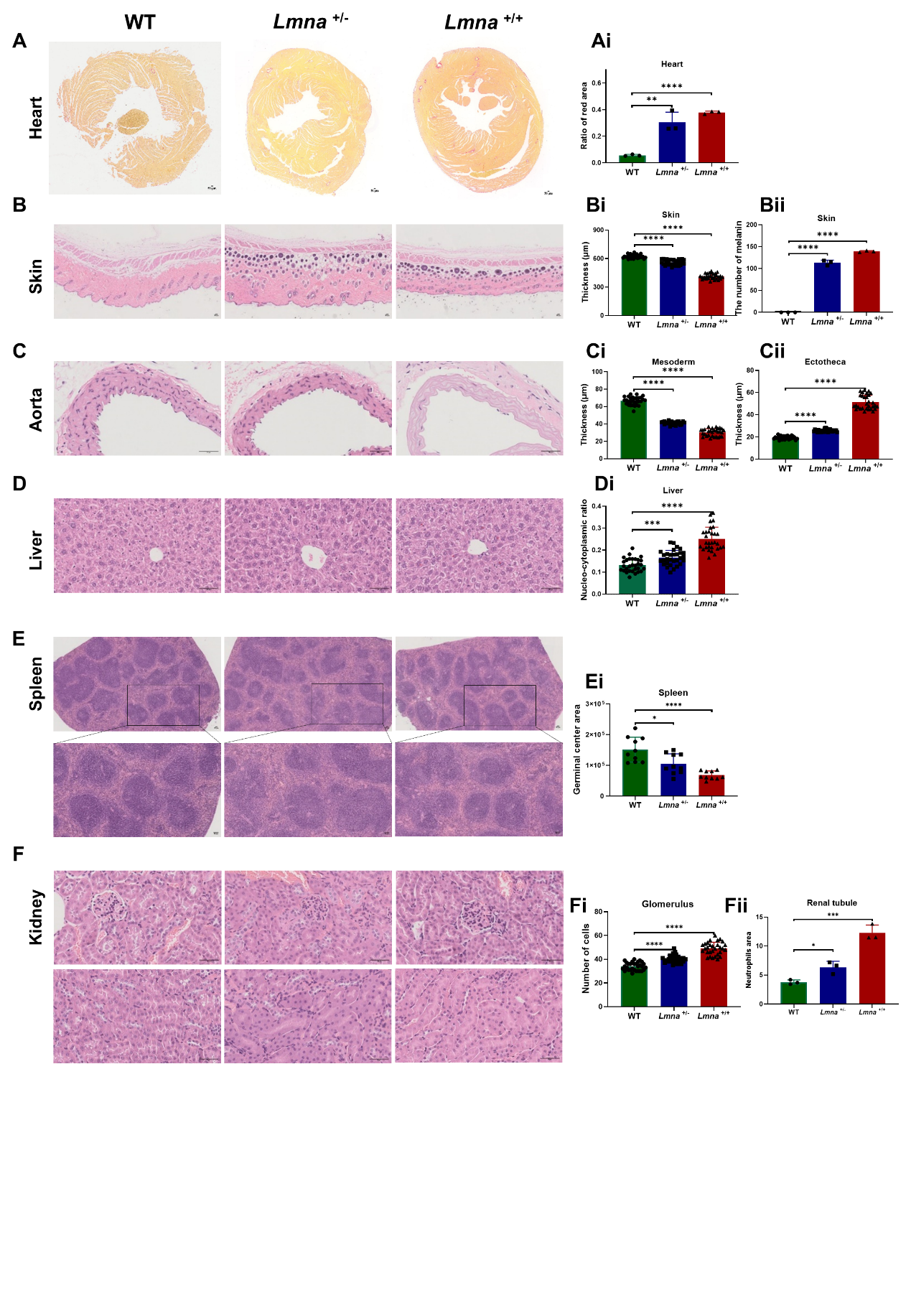


**Figure S2. Quantitative analysis of HE staining in different organs from wild-type (WT), heterozygous** (*Lmna*^G608G/+^) **and homozygous** (*Lmna*^G608G/G608G^) **mice.** (A) Paraffin section of heart tissue stained with Picro-Sirius Red. (Ai) Quantification of the ratio of red area in heart tissue. (B) Paraffin section of skin tissue stained with HE. Measurement of skin thickness (Bi) and number of melanin cells (Bii). (C) Paraffin section of aorta tissue stained with HE. Measurement of tunica media thickness (Ci) and tunica adventitia thickness (Cii). (D) Paraffin section of liver tissue stained with HE. (Di) Quantification of nucleocytoplasmic ratio in liver tissue. (E) Paraffin section of spleen tissue stained with HE. (Ei) Quantification of germinal center area in spleen tissue. (F) Paraffin section of kidney tissue stained with HE. Quantification of the number of cells in the glomerulus (Fi) and renal tubule (Fii). (using student t-test for analysis, ** stands for *p* < 0.01, *** stands for *p* < 0.001, **** stands for *p* < 0.0001).

**
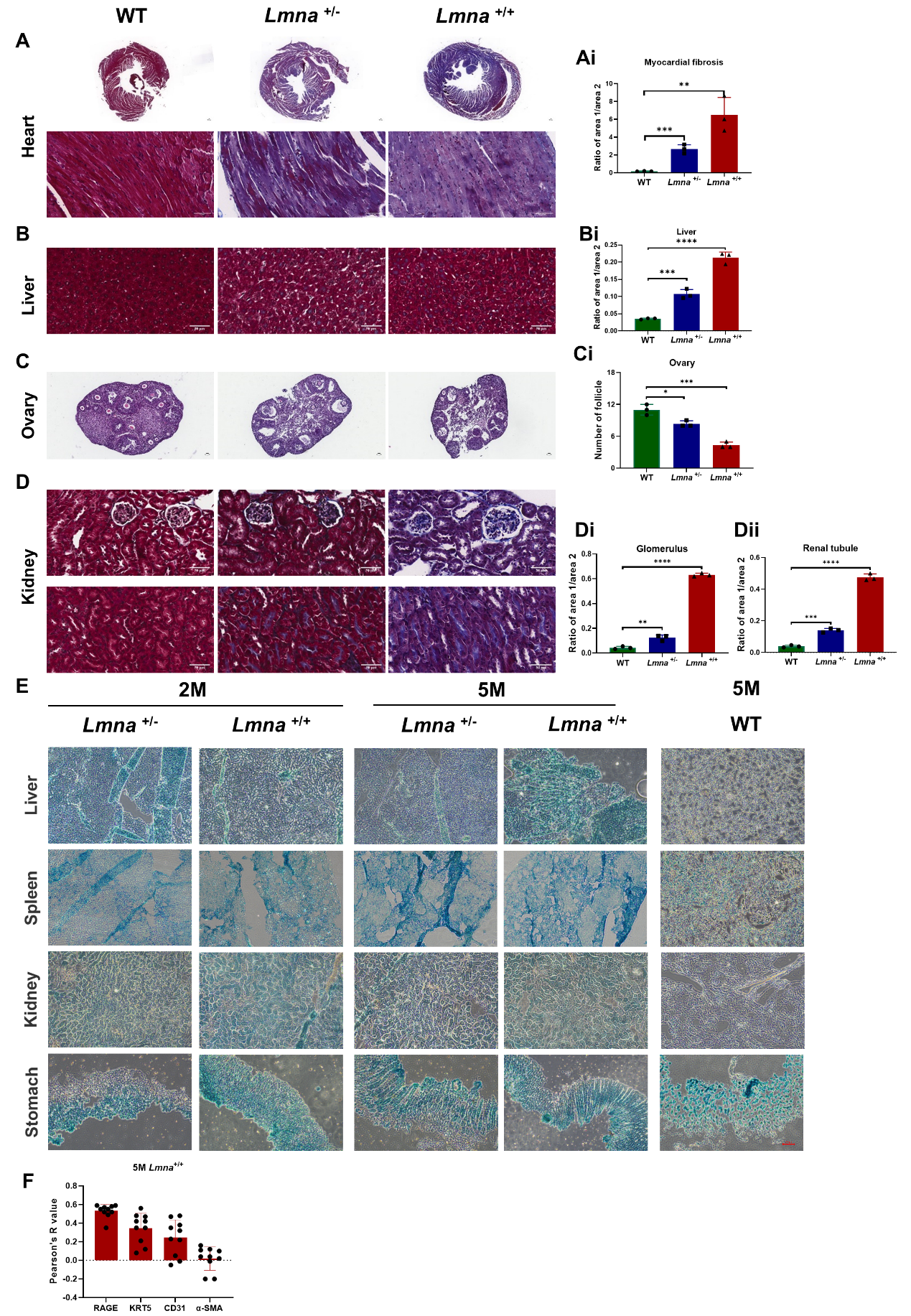
**

**Figure S3. Quantitative analysis of Masson trichrome staining and SA-β-Gal staining in different organs from wild-type (-/-), heterozygous (+/-), and homozygous (+/+) mice.** (A) Paraffin section of heart tissue stained with Masson trichrome reagents. Measurement of myocardial fibrosis in heart tissue (Ai). (B) Paraffin section of liver tissue stained with Masson trichrome reagents. Measurement of hepatic fibrosis in liver tissue (Bi). (C) Paraffin section of ovarian tissue stained with Masson trichrome reagents. Quantification of the number of mature follicles in the glomerulus (Ci). (D) Paraffin section of kidney tissue stained with Masson trichrome reagents. Measurement of glomerular fibrosis (Di) and renal tubule fibrosis (Dii) in mice with different genotypes. (E) SA-β-Gal staining in liver, spleen, kidney, and stomach. (F) Quantitative analysis of Pearson's R value show colocalization level with the expression of Progerin in the lung of wild-type (-/-) and homozygous (+/+) mice at 5 months. (using student t-test for analysis, ** stands for *p* < 0.01, *** stands for *p* < 0.001, **** stands for *p* < 0.0001).
